# Tissue-Engineered Models of the Human Brain: State-of-the-Art Analysis and Challenges

**DOI:** 10.3390/jfb13030146

**Published:** 2022-09-09

**Authors:** Giulia Tarricone, Irene Carmagnola, Valeria Chiono

**Affiliations:** 1 Department of Mechanical and Aerospace Engineering, Politecnico di Torino, Corso Duca Degli Abruzzi 24, 10129 Turin, Italy; 2 PolitoBioMedLab, Politecnico di Torino, Corso Duca Degli Abruzzi 24, 10129 Turin, Italy; 3 Interuniversity Center for the Promotion of the 3Rs Principle in Teaching and Research, Centro 3R, 56122 Pisa, Italy; 4 Nanobiointeractions & Nanodiagnostics, Istituto Italiano di Tecnologia (IIT), Via Morego 30, 16163 Genova, Italy; 5 Department of Chemistry and Industrial Chemistry, University of Genova, Via Dodecaneso 31, 16146 Genova, Italy

**Keywords:** brain model, iPSCs, tissue-engineered models, 3D bioprinting, porous scaffold

## Abstract

Neurological disorders affect billions of people across the world, making the discovery of effective treatments an important challenge. The evaluation of drug efficacy is further complicated because of the lack of in vitro models able to reproduce the complexity of the human brain structure and functions. Some limitations of 2D preclinical models of the human brain have been overcome by the use of 3D cultures such as cell spheroids, organoids and organs-on-chip. However, one of the most promising approaches for mimicking not only cell structure, but also brain architecture, is currently represented by tissue-engineered brain models. Both conventional (particularly electrospinning and salt leaching) and unconventional (particularly bioprinting) techniques have been exploited, making use of natural polymers or combinations between natural and synthetic polymers. Moreover, the use of induced pluripotent stem cells (iPSCs) has allowed the co-culture of different human brain cells (neurons, astrocytes, oligodendrocytes, microglia), helping towards approaching the central nervous system complexity. In this review article, we explain the importance of in vitro brain modeling, and present the main in vitro brain models developed to date, with a special focus on the most recent advancements in tissue-engineered brain models making use of iPSCs. Finally, we critically discuss achievements, main challenges and future perspectives.

## 1. Introduction to Brain Anatomy and Pathology 

### 1.1. Brain Tissue Composition and Structure

Together with the spinal cord, the brain is part of the central nervous system (CNS). It is one of the softest tissues of the human body, mechanically protected by the skull [1]. The brain can be considered a multiscale structured organ from a temporal and spatial point of view, and has the capacity to elaborate different types of inputs (molecular, cellular, neuronal) [2]. Mechanical properties that are involved in neuromechanical signaling deeply influence brain development, physiology and pathology [1]. The main cellular components of the brain are neurons and glial cells. Neuron functions include processing, information storage and transmission of communication signals through chemical or electrical synapses [3]. Astrocytes, microglia and oligodendrocytes constitute 19–40%, 10% and 45–75% of all glial cells, respectively; the remaining cells are NG2 [4,5]; or oligodendrocyte precursor cells (OPCs) [6,7] (Table 1).

Despite representing only 10–20% of the brain volume [18], the brain extracellular matrix (ECM), plays a fundamental role in ensuring proper brain function, contributing to its normal physiology.

The glycoproteins present in the brain ECM, such as laminin, are mainly produced by neurons and glial cells [19]. Barnes et al. [20] reported that, unlike the majority of soft peripheral tissues, brain has a non-fibrillar structure, with low stiffness and an elastic modulus ranging from 0.1 to 1 kPa [21]. Indeed, the main components of brain ECM are glycosaminoglycans (GAGs), including hyaluronic acid (HA) and proteoglycans [22]. GAGs and proteoglycans are highly hydrophilic with high negative charge densities, conferring a high degree of hydration to the brain tissue. Interestingly, due to its proteoglycan-based composition, the brain ECM is able to easily withstand changes in volume [1].

Vecino and Kwok [23] described the properties and functions of the brain ECM. The ECM guarantees direct structural support to neural cells, and allows their anchoring and their organization into different brain regions. Furthermore, brain ECM regulates intercellular communication [24] and, consequently, cellular activities. Hence, the brain ECM can be considered a biological scaffold that deeply influences the biomechanical properties of the brain through the regulation of cell–ECM interactions [25]. Any changes in ECM composition and mechanotransduction can thus reduce the regenerative capacity of the brain [26], while imbalances among ECM molecules can trigger the immune response, leading to inflammation and ECM remodeling [27].

In addition to the brain ECM composition, its structural organization comprises three different layers: (1) the basement membrane (basal lamina); (2) the perineuronal nets and (3) the neural interstitial matrix.

The basement membrane is organized into convex-shaped nanostructures [28]; it separates endothelial cells from neurons and glial cells, and contributes to vessel formation and blood–brain barrier (BBB) integrity [29]. Its main components are represented by collagen IV, laminins and heparan sulfate proteoglycans (HSPGs) [30].

The perineuronal nets have a microfibrous structure with no specific orientation [31], and differ in composition compared to the basement membrane, as they are made of chondroitin sulphate proteoglycans (CSPGs), hyaluronan and tenascin [32]. Important functions of the perineuronal nets are to control synaptic plasticity and prevent neurons from oxidative stress [33,34].

Finally, the neural interstitial matrix components are proteoglycans, hyaluronans, tenascins and linking proteins, with additional small amounts of fibrous proteins and adhesive glycoproteins [35] that are not linked to the basement membrane or the perineuronal nets.

The presence of BBB, which is fundamental for cerebral homeostasis, increases brain peculiarity and complexity. The BBB is part of the neurovascular unit, and its principal role is to allow communication between the CNS and the periphery. The BBB regulates molecule trafficking and prevents the entry of toxins and harmful substances into the brain [36]. BBB structure comprises many cell types, including brain microvascular endothelial cells (BMECs), pericytes, astrocytes and a non-cellular component, which is the basement membrane [37]. BMECs are responsible of the high selectivity of the BBB due to their tight junctions [38], while pericytes are involved in the constitution and maintenance of the BBB. This last function is also carried out by astrocytes, which communicate with BMECs and pericytes through their endfeet and contribute to the selective transport of ions and water [11]. The basement membrane of the BBB, mainly composed of collagen IV, laminin, nidogen and perlecan, ensures adequate vascular function, together with other cellular components [37]. Due to the high selectivity of this barrier, there are many difficulties in delivering drugs to the CNS, and therapeutic strategies able to cross the BBB and reach the brain are under study [39]. Many brain pathologies damage the integrity of the BBB, and this in turn may cause neural disfunctions and degeneration [40]. Hence the BBB represents a key part of the brain structure in studies focus on the therapeutic treatment of brain diseases.

### 1.2. Brain Tissue Alterations under Pathological Conditions

Brain pathological conditions can be classified into five main categories: brain traumas, cerebrovascular injuries, brain tumors, neurodegenerative disorders and psychological conditions. All these conditions may cause changes in terms of both brain cells and ECM composition.

In brain pathologies, astrocytes are in an active inflammatory state, and their role is critical in many brain disorders [41]. For example, in Alzheimer’s disease (AD), astrocytes contribute to the accumulation of amyloid-β [42] and increase in inflammation [43]. Moreover, many pathologies can lead to defects in glutamate and Ca^2+^ signaling, and the inflammatory state may arise as a secondary effect, such as in Huntington’s disease [44]. Metabolic defects linked to astrocyte activity are also involved in the hyperexcitability state of epilepsy [45]. Astrocytes also become reactive after ischemic stroke, contributing to the formation of glial scars [46] and actively participating to the immune response [47]. Moreover, astrocytes are considered regulators of ischemia [45]; this is because in the acute stage of stroke, their function is important for limiting brain damage and neuroinflammation [48], while in the chronic stages, astrocytes promote axon regeneration and neurological recovery [49].

Microglia activation is also important in determining the outcome of many brain pathologies, and, if persistent, can lead to chronic neuroinflammation [50]. Microglia have various roles depending on brain pathology. For example, microglia activation can be considered the first step in the inflammatory response after ischemic brain injury [51]. During the acute phase of stroke, microglia display a proinflammatory role, but subsequently, these cells guide neurogenesis, repair [52] and remyelination after stroke [53]. In AD, extracellular protein aggregates cause the microglia-mediated release of proinflammatory molecules, leading to neuronal damage [54]. Prolonged activation of microglia is responsible for tumor progression in malignant brain tumors [55]. Finally, psychiatric disorders, such as schizophrenia, bipolar disorder and depression may arise because of defective microglia–neuronal activities [56].

Oligodendrocytes do not actively participate in neuroinflammation, but they are the most damaged brain cell type after ischemia, which causes the loss of myelinated axons [57]. In fact, the differentiation of OPCs contributes to the increasing number of myelinating oligodendrocytes after stroke [58]. The improvement of remyelination could be an interesting therapeutic strategy to promote recovery after traumatic brain injury or stroke [59]. Demyelination is also a characteristic of other neurodegenerative diseases, such as AD and Parkinson’s disease (PD), but it can also be found in psychiatric disorders such as schizophrenia and depression [60].

The activated state of glial cells contributes to changes in the composition of the brain ECM, which plays an important role in the modulation of inflammation in brain pathologies [61]. For example, in traumatic brain injury, due to the activation of metalloproteinases (MMP), ECM components, such as aggrecan, increase, together with HA fragmentation and CSPG accumulation, which are responsible for a more severe inflammatory state [19]. MMP activation also occurs in the initial phase of ischemic stroke, and these enzymes are involved in BBB disruption and, consequently, in edema, hemorrhage and leukocyte infiltration, thus triggering the immune response [62]. BBB disruption allows the entry of molecules into the CNS parenchyma, such as fibrinogen, which can cause vessel occlusion and inflammation [63]. Reactive glia, before creating a fibrotic scar, which is softer than the normal tissue, express keratin sulphate proteoglycans (KSPGs) [64] and CSPGs [65], which have been demonstrated to limit post-injury neuronal and axonal regeneration. Moreover, the upregulation of osteopontin (OPN) after stroke seems to be optimal for neural recovery [63].

The alteration of ECM composition is also a key feature in multiple sclerosis, as it prevents the differentiation of OPCs into mature oligodendrocytes and consequently invalidates remyelination [66]. This process is also compromised by upregulation of laminin-2, which promotes remyelination [67], and fibronectin [68], which suppresses it [69], together with HA and CSPGs [70,71].

In AD, activation of astrocytes and microglia causes the alteration of ECM composition relative to HSPGs, which accumulate in amyloid plaques and neurofibrillary tangles [61]. Furthermore, β-amyloids, in addition to microglia, promote the activation of MMPs, thus leading to BBB disruption and exacerbating neuroinflammatory processes [62].

Epilepsy is characterized by the overexpression of molecules that compromise axonal sprouting and synaptogenesis, such as HA, tenascin, hevin and neuronal pentraxin-2 [72]. Downregulation of MMP-9 is one of the principal factors involved in promoting epilepsy, because the lack of regulation of phosphacan and aggrecan contributes to the chaotic environment within the perineuronal nets, which favors synaptic plasticity and reorganization [72].

The brain ECM also favors glioma invasion [73]. Collagen I levels increase in malignant gliomas, unlike in healthy brain tissue, characterized by low levels of fibrillary proteins [74]. Moreover, the increased expression of some GAGs and HA is associated with glioma progression [75]. Upregulation of proteins such as brevican, tenascin-C and fibronectin has been also observed in gliomas [61]. In addition, increased expression of HSPGs glypican and syndecan-1 is crucial for glioma angiogenesis [76].

Hence, pathological brain conditions show specific alterations in the brain ECM and cell microenvironment, which deserve investigation through exploiting suitable experimental in vitro models, as described and discussed in this review article.

## 2. Introduction to Preclinical Models of Human Brain Tissue

**Figure 1:**
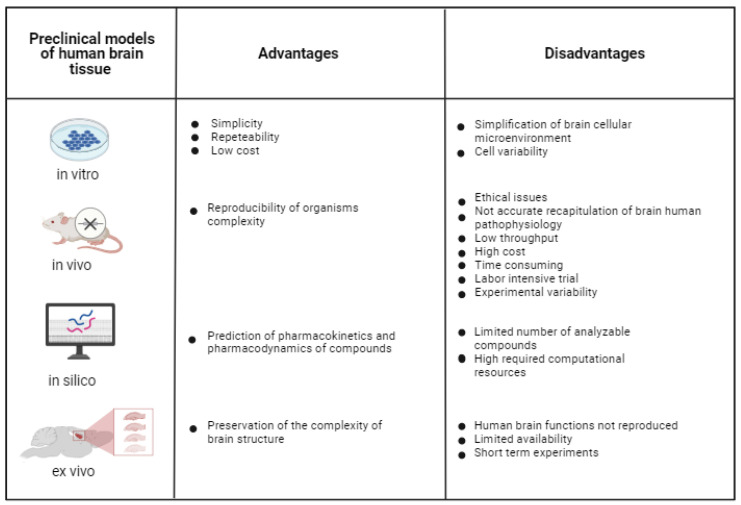
Schematic representation of preclinical models of human brain tissue (in vitro, in vivo, in silico and ex vivo): advantages and disadvantages.

Reliable in vitro models of healthy and pathological brain tissue are under high demand as tools to investigate brain disease pathogenesis, and to discover and preclinically validate new therapeutic approaches [77]. One of the toughest challenges is to develop in vitro brain models that not only include the various brain cell types and ECM components, but also reproduce the complex neural networks that regulate neural functions in healthy and pathological conditions. Many different strategies have been pursued to reproduce the intricate architecture and functionality of the brain, and they can be classified into four different categories: in vitro, in vivo, in silico and ex vivo models (Figure 1).

In silico models complement in vitro, ex vivo and in vivo trials. They make use of computational analysis to investigate physiological/pathological systems and/or the mechanisms of action of pharmacological agents [78]. In silico computational models have the potential to predict the pharmacodynamics and pharmacokinetics of compounds; however, they are limited by the number of analyzable compounds and required computational sources. In silico models will not be treated in this review paper, which is focused on experimental in vitro brain models, but information on in silico models can be found in other recently published reports [79].

In vitro 2D cell models consist of different types of brain cells cultured on 2D culture plates at high cell densities, undergoing spontaneous self-organization. They are widely used during preclinical testing due to their simplicity, repeatability and low cost compared to in vivo models [80]. However, in vitro 2D brain models oversimplify the cellular microenvironment of the brain, as they do not reproduce the complex 3D brain structure [81] and physiological interactions among brain cells [82]. Furthermore, their outcomes are deeply influenced by issues, such as cell variability (e.g., for cell lines), immaturity, limited lifespan and functional development [83].

During preclinical investigations, in vivo animal trials are performed after 2D cell tests, with the aim of assessing drug safety and efficacy, or to investigate brain physiological functions or pathological conditions. Compared to 2D cell cultures, in vivo animal models reproduce the complexity of living organisms, and have been employed for different research purposes, including the study of primary brain tumors [84], brain metastasis [85] and brain ischemia [86]. However, in addition to ethical issues associated with animal experimentation, in vivo models cannot be tightly controlled, unlike in vitro experiments, and they do not offer an accurate recapitulation of human brain pathophysiology due to interspecies differences [87]. Further disadvantages of in vivo animal studies are associated with low throughput, high cost, time-consuming and labor-intensive trials as well variability in results. After the EU ban on animal testing for cosmetic products and ingredients in 2013 (EU Regulation 1223/2009), efforts have been addressed to reduce the use of animals for preclinical validation of drug safety and efficacy, and safety assessment of chemicals, in order to partially or totally replace them with alternative testing protocols, in compliance with the 3Rs principle (Reduction, Replacement, Refinement; Directive 2010/63/EU) [88]. This trend has been confirmed by the recent decision taken by the EU Parliament on 16 September 2021, which aims at the complete replacement of experimental animals with alternative and efficient testing platforms. Considering the current widespread use of animals for preclinical research on the nervous system, the availability of predictive in vitro human brain models is expected to have a high impact on the application of the 3Rs principle.

In this context, even ex vivo brain models, such as brain slides dissected from animals, typically from rodents [86], offer the advantage of preserving the complex brain structure; however, they do not reproduce human brain function, have limited availability and only allow short-term experiments [89].

Due to the above-described limitations of in vivo, ex vivo and in vitro 2D models, the design of 3D human brain models represents a key challenge for improved in vitro reproduction of the complex human brain architecture and functionality. In vitro 3D models of brain tissue allow the study of cell–ECM interactions, cell–cell communication and electrophysiological network properties, filling the gap between 2D cell cultures and animal models [90]. Importantly, in addition to mimicking the 3D cellular environment, 3D models should reproduce brain functionality through mimicking different brain region architectural features and neural networks, as reported by Hopkins et al. [91]. For these reasons, 3D brain models may represent improved tools for the in vitro study of brain disease pathogenesis and efficient drug treatments for brain injuries and diseases.

### 2.1. 3D Brain Tissue Models

#### 2.1.1. Main cell Populations in Brain Tissue Models

Proper selection of cells is fundamental for the engineering of human tissues. The most frequently used cell types are primary cells, immortalized cells and stem cells.

##### Primary Cells and Immortalized Cell Lines

Primary cells directly isolated from tissues or organs are able to reproduce the physiological cellular environment, but their finite lifespan limits their use [92]. Primary cells from the human brain are not easily available, and, for this reason, immortalized cells have been widely used in brain models. Immortalized cells are usually genetically modified and undergo almost unlimited expansion. Immortalized cell lines of neuronal origin have often been employed to study processes such as neural differentiation and axon selection, guidance and growth [82]. The most commonly used immortalized cell lines are SH-SY5Y, PC12, MN9D, N27 and Neuro 2a [93]. SH-SY5Y cells are a subclone of the SK-N-SH neuroblastoma cells, derived from metastatic bone tumor biopsy and widely used for the in vitro differentiation of neural cells [94]. SH-SY5Y cells can be differentiated into cholinergic, adrenergic and dopaminergic neuronal phenotypes, and the resulting cell phenotype depends on the chosen differentiation method [95]. Kovalevich and Langford [94] summarized the most commonly used methods for differentiating SH-SY5Y cells. The addition of retinoic acid (RA) to the cell culture medium induces a strong differentiation of SH-SY5Y into cholinergic neurons and, together with phorbol esters, also drives differentiation of dopaminergic neurons [96]. Treatment with phorbol esters alone, such as 12-O-tetradecanoylphorbol-13-acetate (TPA), or with dibutyryl cyclic adenosine monophosphate (dbcAMP), allows the differentiation of adrenergic neurons [97,98]. Other successful treatments for SH-SY5Y differentiation involve the use of nerve growth factor (NGF) and brain-derived neurotrophic factor (BDNF), which have been proven to be efficient in maintaining the neural phenotype, in combination with RA or TPA [99]. Treatment of SH-SY5Y with cholesterol, vitamin D or insulin enhances neuronal differentiation and survival [100,101,102]. SH-SY5Y cells have been widely employed to understand the molecular mechanisms related to PD [103], AD [104], ischemia [105] and amyotrophic lateral sclerosis [106].

PC12 is another commonly used immortalized cell line derived from a transplantable rat pheochromocytoma of the adrenal medulla [107]. As for SH-SY5Y, treatment with NGF induces the differentiation of PC12 into neuron-like cells [108] and, together with dexamethasone, it also enhances the release of vesicles and neurotransmitters. In fact, PC12 cells have been widely used for endocytosis studies [109] and PD in vitro modeling [110,111].

The MN9D cell line is a fusion of embryonic ventral mesencephalic and neuroblastoma cells, and is one of the most frequently employed cell lines used to study PD. The differentiation of MN9D into dopaminergic (DA) neurons is induced by treatment with GDNF, and the addition of butyric acid causes the cells to acquire electrophysiological properties [112].

The Neuro 2a line has the ability to differentiate into neurons over a short time period: the use of dbcAMP promotes Neuro 2a differentiation into DA cells, enhancing Nurr-related factor 1, tyrosine hydroxylase and DA expression levels, and thus being useful in PD models [113].

##### Stem Cells

Human stem cells represent an important cell source in tissue engineering (TE), particularly when compared to the use of primary cells from animal tissues/organs, which suffers from interspecies differences and ethical problems [90]. The stem cell types employed in regenerative medicine can be divided into three main categories based on cellular source and stem cell potency: embryonic stem cells (ESCs), induced pluripotent stem cells (iPSCs) and adult stem cells (ASCs).

Based on their origin as the inner cell mass of embryo blastocysts, and on their pluripotency, ESCs can be differentiated into each cell type of the human body. Indeed, protocols have been established for their differentiation into dopamine neurons, glial cells, astrocytes and oligodendrocytes [114]. However, their in vivo application as undifferentiated pluripotent stem cells could cause teratoma formation [115]. Moreover, the use of ESCs is also affected by ethical concerns due to their embryonic origin.

A new source of pluripotent stem cells has been made available after Yamanaka and Takahashi’s discovery of human iPSCs [116]. They successfully reprogrammed both mouse and human somatic cells into iPSCs through the expression of four transcription factors via retroviral transduction (OCT3/4, SOX2, KLF4 and MYC). After the delivery of these transcription factors, reprogrammed cells behave as pluripotent stem cells. Fibroblasts are commonly used to generate iPSCs as they are easily accessible, but iPSCs can also be obtained from many other cell types in the body, such as hepatocytes, adipose cells and chondrocytes [117]. iPSCs can now be generated through many different approaches, including the use of integrating or non-integrating viruses (Adenovirus, Sendai virus) [117,118] and non-viral reprogramming methods [119].

iPSC generation has revolutionized the field of regenerative medicine, and their properties make them suitable for brain regeneration and modeling. For instance, Steward et al. [120] highlighted the incredible potential of iPSCs: they are pluripotent, can be cultured and expanded indefinitely, and it is possible to direct their differentiation into any cell type of the body, including nervous system cells. Moreover, they have been successfully used for in vitro developmental studies. The possibility of obtaining iPSCs from somatic cells could allow the modeling of human diseases in vitro from patient-derived cells for personalized medicine research [82,112]. Indeed, iPSCs are ideal for disease modeling and drug screening due to their ability to preserve disease phenotypes [121], providing insight into cellular and molecular pathogenesis of neural diseases, such as Parkinson’s disease and retinal degenerative disorders. Finally, many protocols for iPSC neural differentiation in vitro have been developed. These protocols are specifically for obtaining neurons [122,123], astrocytes [123,124,125] and microglial cells [126,127,128].

Recent attempts have also been performed to directly generate functional neurons from somatic cells by direct reprogramming [129]. This process has been generally mediated by the administration of transcription factors and/or microRNAs to fibroblasts or glial cells to convert them into neurons [130]. Compared to indirect reprogramming of somatic cells into iPSCs, followed by their differentiation into neuronal cells, direct reprogramming does not imply a pluripotent intermediate stage. The main disadvantage of direct reprogramming for use in in vitro modeling is the impossibility of producing a large number of reprogrammed cells without using a large number of somatic cells. On the other hand, once optimized, the process of direct reprogramming could be exploited to obtain the desired cell types in a shorter time period compared to indirect reprogramming. In general terms, another advantage of direct reprogramming is the promise it shows in terms of applying it as an in situ therapy for regenerative purposes, while indirect reprogramming requires in vitro differentiation of cells to avoid teratoma formation in vivo.

#### 2.1.2. Brain Tissue Models

**Figure 2:**
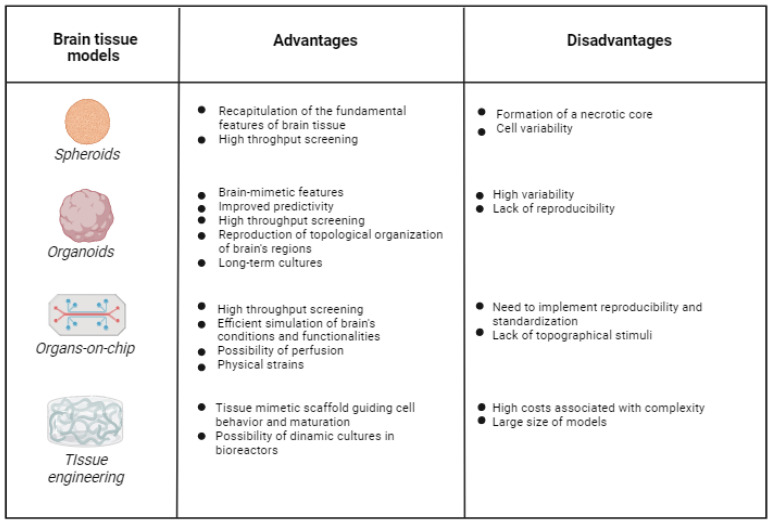
Schematic representation of preclinical in vitro models of brain tissue (spheroids, organoids, organs-on-chip, tissue-engineered models): general advantages and disadvantages.

Different 3D in vitro brain models have been developed, including spheroids, organoids, organs-on-chip and tissue-engineered models (Figure 2). Spheroids can be defined as microtissues in the form of multicellular aggregates [131]. Spheroids, together with organoids, have been the most highly used models for designing 3D in vitro brain models. Additional to favoring cell–cell and cell–matrix interactions, spheroids recapitulate the fundamental features of brain tissues from cell diversity to electrophysiology, ECM production and mechanical stiffness [132]. Neurospheroids have also been exploited to reproduce nervous system pathologies, such as AD [133]. However, one main limitation is the formation of a necrotic core due to gradient supply of nutrients from the shell to the central region of spheroids, which grows proportional to the spheroid size [134]. Hydrogels have been used as matrices supporting the growth of spheroids in attempts to increase permeability [135,136]. However, the necrotic core also formed when hydrogels were used, and could only be avoided by the generation of vascularized spheroids. As well as using hydrogels, spheroid technology may benefit from several bioengineering technologies for reproducible and scalable spheroid preparation, including the use of bioreactors [137].

According to Lancaster and Knoblich, organoids are simplified and miniaturized 3D versions of organs, typically derived from pluripotent or adult stem cells [138,139]. They find application in basic research, biobanking, disease modeling and precision medicine [140]. Despite their well-known advantages, such as brain-mimetic features, improved predictivity compared to 2D cell cultures and high-throughput screening ability [140], they exhibit high variability, due to heterogeneity in the organoid formation process in vitro (in terms of cell organization and fate), and require long-term cultures for cell growth and maturation [141]. hiPSC-based organoids have revolutionized brain-based drug discovery, showing relevant advantages compared to ex vivo models and traditional 2D culture systems. Indeed, brain organoids are efficient at reproducing the topological organization of brain regions in a very similar way to human brain tissue, hence their application in modeling neurodevelopmental disease and in personalized drug screening [142]. The introduction of fused organoids or “assembloid” systems allowed researchers to analyze the interplay between different regions of the brain [143]. Despite their enormous potential, human organoids have limitations attributed to their low size and limited maturation level, namely, failure to reproduce brain development and adult brain tissue [144]. Other problems include their lack of reproducibility and the absence of microglial cells [145]. To address some of the current limitations, organoids could be supported by bioengineered biomimetic hydrogels to better mimic brain tissue, favoring cell differentiation and maturation [146].

Organs-on-chip are 3D cell culture systems within microfluidic channels, more closely mimicking the functions of tissues and organs than 2D cell cultures, spheroids and organoids [147]. Organs-on-chip allow high-throughput screening for drug safety and efficacy in both healthy and pathological models [148]. Brain-on-chip may efficiently simulate chemical, electrical and physical conditions of the brain [149]. Despite their known ability in reproducing certain brain functionalities (e.g., synaptic communication and neural electrical activity [150]), brain organs-on-chip are generally simplified and do not include all brain cell types. Furthermore, several preclinical evaluation tests (e.g., those based on RNA extraction) are more difficult in 3D cell cultures, such as organ-on-chip systems, than in traditional 2D monolayer cultures. Hence, this technology requires further efforts for improved reproducibility as well as standardization [151]. Currently, different types of brain-on-chip (BoC) platforms have been developed for different purposes, and can also be interconnected with other organ-on-chip systems to evaluate interactions between the brain and other organs [152], or they can be integrated with well plates to perform high-throughput analyses [153]. In addition to mimicking healthy nervous tissues, numerous attempts for modeling brain pathologies, such as AD [154] and PD [155], have been pursued, with promising results produced by BoCs. Despite these advantages, Miccoli et al. [156], highlighted the limitations of current organ-on-chips devices. Such models usually do not replicate the complexity of the brain tissue and its pathological conditions, because they allow the reproduction of a specific and localized brain region instead of the entire brain tissue. Despite the fact that stem-cell-based brain-on-chips have been prepared, they suffer from difficulty in stem cell differentiation, as well as poor reproducibility and variability. To this purpose, appropriate optimization of ECM, mimicking the physiological brain environment, could help in directioning cell behavior in BoCs. Furthermore, despite the recent advances in immunotherapy-on-chip [157], most brain-on-chip models lack immune cells, so that inflammatory brain conditions cannot be reproduced. Brain-on-chips can also take advantage of the use of hydrogels: 3D high-content BoCs with hydrogels have been employed to verify neural circuit formation [153] and to recapitulate BBB structure and functions [158,159].

In addition to spheroids, organoids and organs-on-chip, tissue engineering (TE) represents a promising interdisciplinary field with the potential for the realization of complex 3D brain models. By integrating biology with engineering, TE aims at recreating human tissues for regenerative and in vitro modeling purposes [91], exploiting engineered scaffolds (Figure 2). TE models can better mimic the target tissue due to the use of biomimetic scaffolds with tissue-mimetic biochemical and biophysical properties [160], providing control over cell adhesion, morphology, growth, proliferation and differentiation [161]. Scaffolds are temporal ECM-like substrates, suitable for long-term cell cultures, then gradually replaced by cell-secreted ECM [162,163]. Scaffolds can also be enriched with biomolecules, such as growth factors, to provide additional paracrine signaling to cells. TE is rapidly growing due to continuous advances in biomaterial science and scaffold fabrication techniques, as evidenced by the introduction of reproducible and scalable rapid prototyping technologies for scaffold fabrication and 3D bioprinting [164].

TE approaches have been used to develop 3D models of healthy and pathological tissues and organs for the testing of drug delivery, safety and efficacy in the treatment of neurodegenerative diseases [165]. Furthermore, TE allows to develop in-scale rather than miniaturized models [166]. Among TE methods, bioprinting is an additive manufacturing technique that allows the “printing” of cells and ECM-like hydrogels, to recreate tissue/organ structure after a post-printing in vitro culture step [167]. Interestingly, spheroids and organoids could also be exploited as building blocks in the automated bioprinting process of brain models [168].

Brain TE models making use of multiple cell types (obtained from iPSCs) and different 3D substrates (hydrogels and solid/porous/fibrous scaffolds) will be described and discussed in the next paragraphs as a main focus of this review paper.

## 3. Focus on TE Brain Models

### 3.1. General Design Criteria for TE Brain Models

**Figure 3:**
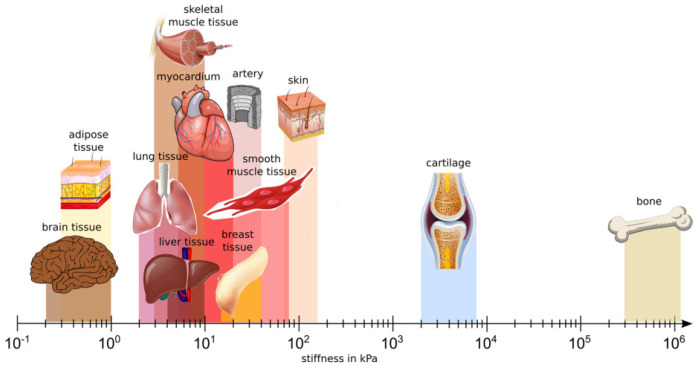
Stiffness of different human tissues. Brain tissue is one of the softest tissues in the human body. From Budday et al. [172].

Scaffolds for brain tissue engineering should mimic brain ECM composition, structure and mechanical properties and satisfy porosity, surface charge and wettability requirements (Table 2) to support cell adhesion, proliferation, differentiation and maturation, which, in turn, drive neuronal functions (assessed by gene and protein expression), neurite outgrowth, folding of the typical brain cortices and tissue functionality [169]. Mammadov et al. reported the target characteristics of scaffolds for iPSC differentiation into brain cells [170]. For example, ECM-like nanofibrous scaffolds mimic the physiological native brain environment, favoring cell–cell and cell–biomaterial interactions. This effect is further reinforced by the use of biomimetic biomaterials provided by bioactive molecules or motifs present in the brain ECM [171]. Brain stiffness is lower compared to other soft tissues, such as cardiac and chondral tissues, and further decreases in pathological conditions with the formation of glial scars [161] (Figure 3). Hence, scaffolds for brain modeling should mimic the low stiffness of the brain.

Interestingly, the use of electrically conductive biomaterials has also been proposed to support neuronal differentiation.

Different brain areas possess specific morphologies and multiscale features [173]. Scaffold composition and design should be tailored to reproduce the heterogeneity and complexity of the brain tissue [174] by mimicking the appropriate ECM components and structural organization of the different anatomical brain regions. For example, as white matter contains aligned and myelinated axonal fibers, and is mechanically anisotropic, scaffolds with oriented geometry should be exploited for its engineering [175]. Moreover, scaffold porosity should be carefully designed to mimic the intrinsic porosity of brain tissue, which is fundamental to ensure the flow of interstitial fluid under existing or induced pressure gradients [176], and to favor cell-specific growth and differentiation [177]. Engineered brain models should not only include neurons, oligodendrocytes and astrocytes, but also microglial cells, in order to reproduce inflammatory pathways in both healthy brain and neuroinflammatory disease models [160]. Further requirements for 3D models reproducing brain compartmentalization and organization are scalability, reproducibility, ease of preparation and cost effectiveness.

**Table 2:** Main scaffold requirements for brain tissue engineering.

Properties	Target Specification/Values	Ref.
Biomimetic composition	Scaffolds should contain proteins (or their peptide motifs) and/or polysaccharides naturally present in the brain ECM or biomimetic with respect to brain ECM composition. Examples include: collagen, laminin, gelatin, fibrin, hyaluronic acid, chitosan, bacterial cellulose, RGD peptide, TATVHL peptide, poly(lysine), poly(ornithine). Surface charge and wettability influence cell behavior on scaffolds. Synthetic polymers, such as poly(lactic-co-glycolic acid) (PLGA) and poly(caprolactone) (PCL), have also been used to improve the stability of scaffolds for brain TE, in combination with natural polymers or adhesive peptides, forming “bioartificial” biomaterials.	[178,179,180,181,182,183,184,185,186,187]
Biomimetic stiffness	Target value of scaffold stiffness is brain tissue stiffness (0.5–14 kPa). Soft gels with moduli <1 kPa were found to enhance neural stem cells (NSCs) differentiation.	[188,189,190]
Biomimetic architecture	Fibrous scaffolds with aligned fibers are suitable for the engineering of white matter, which contains aligned and myelinated axonal fibers, and is mechanically anisotropic. Fibrous scaffolds with randomly oriented fibers have been demonstrated to favor cortical NSC proliferation and differentiation.	[191,192]
Electrical conductivity	Electrically conductive scaffolds containing electrically conductive polymers (e.g., polyaniline (PANi), poly(3,4-ethylenedioxythiophene) (PEDOT), and polypyrrole (PPy)) or polymer composites (e.g., containing graphene or carbon nanotubing (CNT)) can enhance neural regeneration.	[188,193]
Porosity	Scaffold pore size and porosity degree influence cell infiltration and tissue ingrowth, as well as scaffold mechanical properties and degradation rate. The optimal range for scaffold porosity should be compatible with the size of an adult stem cell (20 µm approximately). Small pores (<100 um) favor stem cell adhesion and local niche formation, while larger pores (120 um) are ideal for nutrient and oxygen delivery. Additionally, small pores reduce penetration of morphogenetic factors, influencing cell differentiation.	[174,194]

### 3.2. Relevant 3D Brain TE Models Using iPSCs

Based on the state-of-the-art analysis, the most important 3D platforms used for neural tissue engineering have been grouped into four main categories: hydrogels, 3D bioprinting constructs, decellularized scaffolds and fibrous scaffolds. Each of them presents some peculiarities, which can be implemented by the combination of different 3D platforms to create a more complex structure. In this section of the review, we describe, briefly, each platform (summarized in Table 3), with a special focus on their highlights.

Hydrogels represent one of the most frequently used platforms in brain tissue modeling [195]. They are hydrophilic networks with high water absorption ability and can be classified based on their source, polymeric composition, type of crosslinking, physical appearance and electrical charge [196]. Cell growth benefits from their outstanding physical and chemical properties, providing the ideal 3D microarchitecture for neural regeneration. The main advantages of hydrogels concern their maximum flexibility and ease in modifying material characteristics to adapt to neural tissue heterogeneity. Hydrogels positively influence cell orientation on the scaffold due to their physical guidance ability (mechanical stress, cytoskeletal dynamics, mechanosensing) and cell behavior and differentiation through molecule incorporation for localized release (neurotrophic factors and drugs). For these reasons, hydrogels can be employed alone or they can be implemented into other 3D neural models based on different scaffold types.

The use of hydrogel is also fundamental in the 3D bioprinting approach, representing a key element in the bioink development. According to Murphy et al. [197], 3D bioprinting allows the production of 3D constructs through a layer-by-layer deposition of bioinks loaded with cells. Computer-aided design (CAD) allows the preparation of constructs with reproducible structures. Not surprisingly, the main advantages of 3D printing are personalized design and precise manufacturing. Bioink, cells and biochemical factors are the main players in the 3D bioprinting of brain models [198]. Depending on the bioprinting approach, bioink can consist of cell-embedded hydrogel (scaffold-based approach), or cells alone (scaffold-free bioinks). In the latter, cells are printed at high concentration (e.g., in the form of spheroids), and, during the post-processing culture stage, they deposit their own ECM, ensuring structural support and cell maturation in the developing tissue [199]. In the scaffold-based approach, bioinks are based on cell-containing natural, synthetic or hybrid hydrogels, and must fulfill different requirements: biocompatibility, support of cell cultures, biodegradability, permeability to oxygen and nutrients, injectability, and printability (depending on shear thinning behavior, viscoelasticity and gelation ability) [200].

In the field of 3D bioprinting, pre-printing and post-printing cell differentiation methods can be used. In the first approach, iPSCs are differentiated before being embedded into the bioink for 3D bioprinting. Pre-printing differentiation methods offer the advantage of controlling the position and the density of differentiated cells in the resulting construct. In the post-printing approach, differentiation is induced after construct fabrication. Drawbacks include the long time period needed for stem cell differentiation, which may cause the loss of integrity of the initial printed structure, the inability to control the relative distribution and density of differentiated cells and to purify them from undifferentiated cells. Other issues concerning the bioprinting of iPSCs based bioinks are associated with the need to preserve cell viability, which may be compromised by shear stresses and single-cell dissociation during processing, considering that iPSCs are of embryonic and epithelial nature and tend to aggregate into clusters or colonies [201].

Decellularized scaffolds can be obtained after the removal of cellular components from tissues or organs. The decellularized ECM resembles the native tissue composition and influences cell behavior in terms of cell growth and differentiation [202]. Chemical, biological and mechanical methods can be employed to derive decellularized matrices [203].

Relating to tissue engineering applications, decellularized scaffolds offer important advantages [204]: ECM structure and composition favors recellularization and cell remodeling. In the field of brain modeling, decellularized matrices support NSC attachment and proliferation, since they have low immunogenicity and are biologically recognizable, and either allow the retainment of cell stemness or induce differentiation [205], as reported for PC12 cells [206].

Fibrous scaffolds have been commonly used in TE as they mimic the structure of the brain ECM. One of the main advantages of fibrous scaffolds is the possibility of controlling fiber orientation, with the preparation of random or aligned fibrous scaffolds. They offer a high surface-to-volume ratio, consequently favoring cell adhesion, proliferation and differentiation [207]. Fibrous scaffolds are produced through a range of techniques, such as self-assembly, template synthesis, phase separation and electrospinning, making use of both natural and synthetic polymers [208,209].

#### 3.2.1. Hydrogel-Based Models

Many natural iPSC-containing hydrogels have been developed for neural tissue engineering and functionalized with proteins or electrically conductive materials to improve scaffolds performances and cellular behavior.

Kuo et al. [210] utilized an alginate-based inverted colloidal crystal (ICC) scaffold, containing poly(γ-glutamic acid) (γ-PGA) and functionalized with TATVHL peptide, to investigate its influence on iPSC differentiation towards neurons. γ-PGA improves antibacterial activity and has low immunogenicity [211], while TATVHL peptide enhances neural differentiation [212]. ICC scaffolds were composed of inverted monodispersed colloidal particles, organized to form long-range crystals [213]. Such scaffolds allowed cell colonization and growth, as well as the transfer of nutrients, due to their controlled interconnected porosity and topology [214,215,216]. The presence of TATVHL peptide increased iPSC adhesion and viability, which was higher than 90%. Finally, the differentiation of iPSCs towards neurons was shown by evaluating β-III-tubulin expression [217].

Zhang et al. [218] produced a 3D brain model to investigate modifications in neural cell migration and maturation in neurodevelopmental disorders and, specifically, in Rett syndrome, a genetic disease affecting children and linked to a mutation in the X-linked gene methyl-CpG-binding protein 2 [219]. Density gradient multilayer polymerization [181] was used to prepare methacrylated HA (HAMA) hydrogels. Hyaluronic acid was selected to produce hydrogels with brain mimetic composition; methacrylation degree influenced hydrogel crosslinking and stiffness, in turn affecting cell response [220]. The authors used iPSC-derived neural progenitor cells (NPCs) differentiated into astrocytes and neurons. Cellular identity was confirmed through the evaluation of the expression of glial fibrillary acidic protein (GFAP) and S100 calcium-binding protein β (S100β) for astrocytes [221], and β-III-tubulin (Tuj1) and microtubule-associated protein 2 (MAP2) for neurons. Migration of NPCs towards neurons and astrocytes was evaluated at time 0 and after 3 days. When astrocytes or neurons were seeded on the bottom hydrogel surface and NPCs were cultured on the top hydrogel surface, a consistent migration of both neurons and astrocytes to NPCs was observed. In the opposite arrangement, with NPCs on the bottom hydrogel surface and astrocytes or neurons on the top hydrogel surface, no cell migration was observed. Hydrogels were also exploited to assess defective migration among NPCs derived from Rett syndrome patients. Furthermore, the 3D hydrogels favored reductions in neurite outgrowth and synapsis number in Rett syndrome neurons, and accelerated neural differentiation of human iPSC-derived NPCs. Such hydrogels supported neuronal over glial differentiation, and accelerated the maturation of neurons compared to 2D culture.

Another hyaluronic-based hydrogel scaffold was obtained by Shin et al. [183]. The authors developed electroconductive HA hydrogels incorporating single-walled carbon nanotubes (CNT) and PPy to test the efficiency of differentiation of both human fetal neural stem cells (hfNSCs) and hiPSC-NPCs. CNTs and PPy were chosen as they are electrically conductive materials characterized by low impedance and high charge storage capacity [222]. CNTs were first dispersed in a cathecol-functionalized HA (HA-CA) pre-gel conjugate solution, after a short sonication, under specific temperature conditions. Gelation of HA-CA with electroconductive materials and oxidative polymerization of pyrrole (Py) were performed with the use of the oxidizing agent sodium periodate (NaIO_4_). These electroconductive hydrogels showed enhanced electroconductive properties after the incorporation of CNTs and PPy. hFNSCs encapsulated into hydrogels were found to efficiently differentiate into neurons (Tuj1), astrocytes (GFAP) and oligodendrocytes (CNPase) within 5 days, depending on PPy and CNTs concentrations. Otherwise, hiPSC-NPCs expressed typical neuronal markers (β-III-tubulin and MAP2) 7 days after encapsulation into the hydrogels, and neural differentiation was also demonstrated by upregulated calcium channel expression and enhanced calcium influx. However, iPSC-NPCs did not differentiate into different brain cell types.

Collagen-hydrogel-based models were developed by Pietrucha et al. [184], including neurons, oligodendrocytes and astrocytes, for the study of physiological and pathological brain conditions. Collagen (COL) (especially type I) is the most abundant component of ECM of body tissues, and has been largely employed to prepare brain models. The authors evaluated the effects of different collagen modifications on the growth and differentiation of hiPSC-NPCs. They functionalized collagen with chondroitin sulphate (CS) or 2,3-dialdehyde cellulose (DAC) crosslinking agent. CS was chosen because of its capacity to support neural stem cells, while DAC was selected because of its biocompatibility, degradation properties and low toxicity [223]. COL-CS 3D scaffolds were prepared by a two-stage process, consisting of multiple freeze drying steps followed by EDC crosslinking [224]. COL-DAC scaffolds were obtained by following three steps, consisting of the preparation of DAC by selective oxidation of cellulose, the fabrication of 3D Col sponge shapes and crosslinking in a solution containing DAC [223]. iPSC-derived NPCs were able to penetrate inside the spongy structure of both scaffold types. Scaffolds enhanced hiPSC-NPC proliferation, and allowed different distributions of cells, favoring the formation of active proliferating clusters of cells or a monolayer of cells with flattened and branched morphology. Differentiation of NPCs into neural cells (β-tubulin-III expression), astrocytes (GFAP expression) and oligodendrocytes (platelet-derived growth factor-α and galactocerebroside expression) was demonstrated after 6 days of culture. Results confirmed the ability of these hydrogels to support neural differentiation of iPSCs and the realization of a more complete brain model, including different brain cell types.

**Figure 4:**
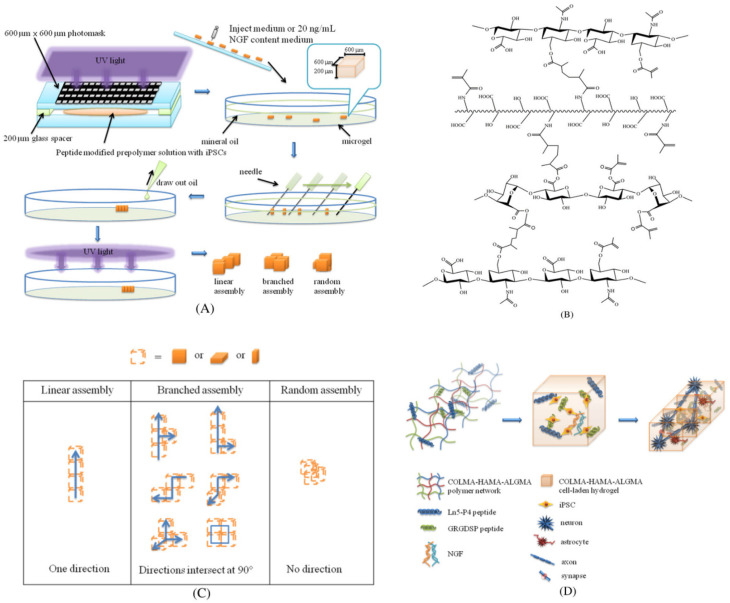
Schematic system of photocrosslinking and experimental setup (**A**), macromolecular structure of COLMA/HAMA/ALGMA microgel (**B**), microgel molecular structure (**C**) and internal structure of self-assembled microgel building blocks (**D**). Reproduced with the permission of Kuo et al. [182].

Many studies reported the development of hydrogels through a combination of different natural materials. Kuo et al. [182] engineered a hydrogel composed of methacrylated collagen (COLMA), HAMA and methacrylated alginate (ALGMA), and functionalized with GRGDSP and Ln5-P4 (Figure 4). GRGDSP is a fibronectin peptide sequence favoring cell adhesion, while Ln5-P4 is involved in signaling pathways that stimulate cell survival [225]. The hydrogel was obtained through blending followed by photocrosslinking under a patterned mask. One of the most interesting characteristics is that COLMA/HAMA/ALGMA hydrogel can aggregate to form linear, branched or random structures. The linear structure was the most appropriate for neural tissue engineering, and it increased after choosing the appropriate ratio among COLMA, HAMA and ALGMA (1:2:1) microgels, of which assembly by hydrophilic attraction was obtained in hydrophobic medium. The functionalization with GRGDSP/Ln5-P4 increased the entrapment efficiency of iPSCs into the hydrogel and an appropriate ratio between the two proteins (3:1) influenced neuronal differentiation. Moreover, after induction of NGF, the percentage of β-III-tubulin-positive cells reached 98%. From these results, it is possible to conclude that COLMA/HAMA/ALGMA hydrogels functionalized with GRGDSP/Ln5-P4 favored neural differentiation of iPSCs, and they can be used for brain regenerative purposes. However, this kind of scaffold did not allow differentiation into other brain cell types.

Polymerized high internal phase emulsion (polyHIPE) materials are solid porous materials widely used for tissue engineering applications. They offer many advantages, such as stability, ease of manufacture and increased nutrient diffusion, due to their characteristic internal structure [226,227].

Polyethylene glycol diacrylate (PEGDA) polyHIPE scaffolds produced by thiol–ene photopolymerization showed biomimetic mechanical properties compared to the brain tissue, and supported the attachment and expansion of hiPSC-NPCs in vitro [228]. Laminin-coated PEGDA and control trimethylolpropane triacrylate (TMPTA) polyHIPE scaffolds were prepared. hPSC-NPCs seeded on scaffolds showed significantly higher viability on laminin-coated PEGDA scaffolds than on TMPTA scaffolds during 14 days of culture. Moreover, both scaffolds triggered the downregulation of early neuroectodermal lineage markers (PAX6, SOX1) [229] and the upregulation of glial cell markers after 14 days of culture in NPSC maintenance medium. Interestingly, stiffer TMPTA scaffolds favored glial over neural differentiation of hiPSC-NPCs, as demonstrated by the downregulation of neuronal markers β-III-tubulin and MAP2, and upregulation of the astrocyte marker GFAP. Additionally, stimulation with glutamate increased calcium concentration levels within differentiated cells, and cells differentiated on laminin-coated PEGDA polyHIPE scaffolds expressed glutamatergic receptors. To conclude, spontaneous calcium activity increased within laminin-coated PEGDA polyHIPE scaffolds.

Kuo et al. [187] prepared hybrid polyacrylamide–chitosan (PAAM–CH) ICC scaffolds to direct iPSC differentiation into neurons and prevent the formation of glial scar, which represents an obstacle in neural regeneration. PAAM is an acrylamide-derived polymer and its properties, such as stability, non-immunogenicity, biocompatibility and viscoelasticity, make it ideal for tissue engineering [230]; however, PAAM hydrogels are not cell adhesive. Instead, CH supports cell adhesion, and can be functionalized with proteins or bioactive peptides [231,232]. PLGA nanoparticles (NPs) are also employed for many applications in regenerative medicine, for their biodegradability, biocompatibility and controlled drug release ability [233]. Polystyrene microspheres were embedded into PAAM–chitosan hydrogel, followed by immersion in tetrahydrofuran and, then, in acetone for the production of the inverted replica. PLGA NPs prepared by an emulsion-diffusion method were injected into PAAM–chitosan ICC scaffolds, followed by further injection of TATVHL peptide solution. Increased cell adhesion was favored by the surface roughness imparted by PLGA NPs and the presence of TATVHL peptide. iPSCs showed enhanced neural differentiation (as assessed by the presence of β-III-tubulin-positive cells) after 3 days culture, while astrocytes formation was inhibited. Interestingly, excessively high concentration of PLGA NPs limited cell differentiation.

Table 4 summarizes the main examples of brain tissue models based on hydrogel scaffolds described in the paragraph.

#### 3.2.2. Biofabricated Brain Models

In the field of brain tissue engineering, 3D printing may generate more reproducible and controlled models. The 3D platform realized by Gu et al. [234] is a key novelty in the field of 3D printed brain constructs. They designed a novel bioink for bioprinting, based on alginate, carboxymethyl chitosan (CMC) and agarose, containing human neural stem cells (hNSCs). Agarose was exploited to adjust the viscosity of the bioink and improve print fidelity, while an alginate and CMC mixture influenced cell response. Indeed, CMC concentration affected gel porosity, permeability and cell viability. On the other hand, after bioprinting, the bioink was crosslinked by calcium ions exploiting the ion crosslinking ability of the alginate component. After crosslinking, the bioink reached a compressive modulus of about 7.5 kPa, while indentation modulus was of about 4.75 kPa, and it decreased to 0.8 kPa after 13 days. The bioink showed similar stiffness to that of brain tissue (which is in the 0.5–14 kPa range) [235]; furthermore, it was highly homogeneous (differentiation of hNSCs into neurons and glial cells occurred 10 days post-printing). Expression of TuJ1, GFAP and OLIGO2 indicated differentiation into all brain cell types, and the increase in the levels of GABA neuronal marker was also observed. Neurons showed spontaneous calcium spikes and a calcium response induced by bicuculline. The authors suggested the usefulness of this platform to study neurodevelopment and brain diseases and for preclinical drug screening.

The same group published another bioprinted brain model [236] using the previously designed bioink in combination with iPSCs. After printing, scaffolds with iPSCs were maintained in culture using a specific medium for stem cell proliferation and self-renewal. Cell viability was 20% at day 1 after printing, while it reached 95% at day 7 and 80% at day 11. iPSC proliferation resulted in spheroid formation by day 7 of culture, and reached its maximum after 9 days of culture. Moreover, iPSCs maintained their characteristic pluripotent state (assessed by the expression of markers of pluripotency, as OCT4, SOX2, TRA-1-60 and SSEA4) after 10 days of culture. Expression of markers of all three lineages (endodermal, mesodermal and ectodermal) indicated that cells had the potential to differentiate into different cell types. Then, a change in medium composition via the addition of BDNF allowed iPSCs differentiation into neural and glial phenotypes 20 days post-printing. As for the previous model, spontaneous and bicuculline-induced calcium responses were evaluated. The strength of this model is due to its ability to differentiate iPSCs within the bioprinted constructs, inducing their conversion into functional neural cells for 3D neural tissue formation.

**Figure 5:**
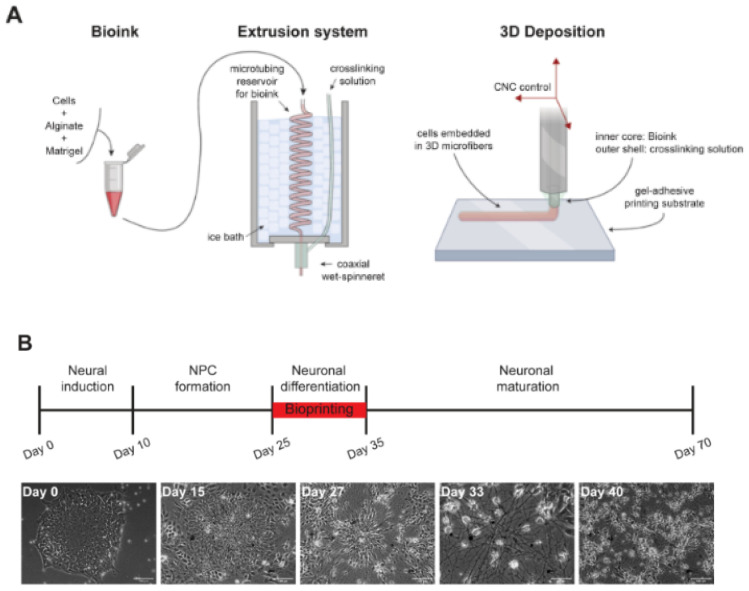

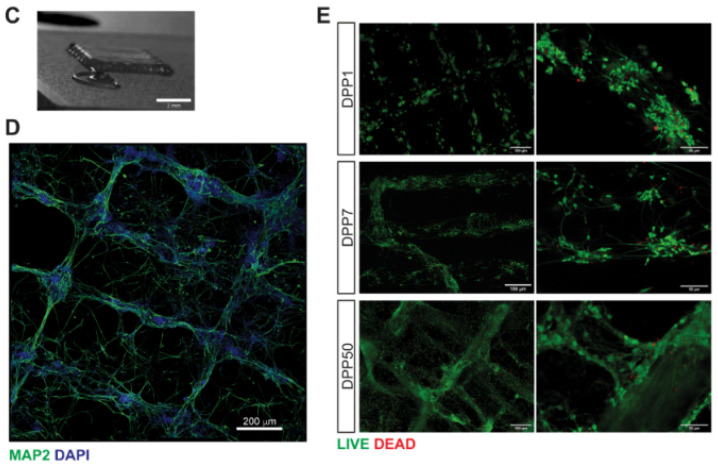
Schematic representation of bioprinting system (**A**), iPSC neural differentiation protocol (**B**), bioprinted construct (**C**), MAP2-stained bioprinted cells at DPP7 (scale bar = 2 mm) (**D**) and live and dead staining at different days post-printing (scale bar = 150 µm for left panels; scale bar = 50 µm for right panels (**E**). From Salaris et al. [237].

Salaris et al. [237] prepared 3D constructs containing iPSC-derived cortical neurons and glial cells by a properly modified extrusion-based printer (Figure 5). They differentiated iPSCs into cortical neurons in vitro by modifying the protocol described by Shi et al. [238], with initial dual SMAD inhibition and subsequent blockage of Hedgehog signaling by cyclopamine. An alginate-based bioink was prepared following the protocol by Yuk et al. [239]. After 4 weeks of iPSC differentiation, cells were suspended in a mixture of Matrigel and alginate (1:1 weight ratio; 2% alginate concentration), forming the bioink. The bioprinted grid-shaped geometry consisted of two alternating layers of perpendicular microfibers with controlled diameter and thickness to easily provide cells with nutrients, and the effect of different cellular densities on cell behavior was analyzed. The authors verified the stability of the constructs over time and evaluated cell viability at different days post-printing (78% at 1 day post-printing, 71% at 7 days post-printing, 68% at 50 days post-printing). Cells in bioprinted constructs expressed both neuronal (especially TRB1 for mature cortical neurons) and glial (GFAP) markers, while mature cortical neurons were maintained in neuronal differentiation medium up to 70 days. The 3D bioprinted constructs also displayed calcium activity at 7 days post-printing. According to the authors, this construct could be exploited to model healthy and pathological brain tissue.

Abelseth et al. [240] first prepared a fibrin-based bioink containing hiPSC aggregates; printing was performed using RX1 printer and lab-on-a-printer (LOP) technology. Fibrin is a protein hydrogel, naturally forming during coagulation and widely employed in tissue engineering to influence cell behavior in terms of adhesion, proliferation and differentiation [241,242]. Abelseth et al. developed a bioink composed of fibrinogen and alginate and also containing a mixture of chitosan, calcium chloride, thrombin and genipin, to ensure printability and stability. The process started with thrombin-mediated cleavage of fibrinogen. LOP technology enables rapid switching between different biomaterial bioinks during the printing process and offers many advantages related to cell protection from various forms of shear stress and their effects on cell death and premature differentiation. The bioink was prepared through combining genipin and alginate solutions, followed by their addition to cell/fibrin solution and further printing after crosslinking with a solution of chitosan, calcium chloride and thrombin. The 3D printed neural aggregates were maintained in culture for 41 days, and the 3D printed constructs displayed resistance to degradation. Moreover, at day 17, RA was added to the culture medium to allow differentiation into dopaminergic neurons. High cell viability was confirmed by calcein assay after 10 and 15 days of culture (94% at day 10, 64% at day 15) and by flow cytometry at day 6 (91%). Moreover, immunostaining at day 41 was positive for Tuj1 expression (an early neuronal marker). In conclusion, the bioprinted constructs enhanced neuronal differentiation, as iPSC-derived neural aggregates survived and differentiated over a culture period of 41 days.

Based on this model, Georges et al. [243] prepared a 3D bioprinted brain model encapsulating hiPSC-NPCs into the above-described fibrin-based bioink with drug-loaded microspheres, to verify the efficiency of drug release on a healthy brain tissue. Microspheres were loaded with guggulsterone, an anticancer drug that is known to induce the differentiation of both hESCs and hiPSCs into dopaminergic neurons [244,245]. hiPSCs were first differentiated into NPCs and expanded in culture before encapsulation into the fibrin bioink. Constructs with drug-loaded microspheres, unloaded microspheres and soluble guggulsterone were prepared. NPCs and microspheres were homogeneously distributed in all constructs, and they maintained their characteristic dome shape after printing. High cell viability was observed in all printed structures 1 day post-printing (90%), but the highest viability value was found in 3D constructs containing guggulsterone microspheres after 7 days of culture (95%). Specific neural markers were detected by immunocytochemistry after 15 and 30 days of culture, while the presence of cells expressing glial (GFAP) and oligodendrocyte markers (O4), was assessed by flow cytometry. The 3D tissue expressed markers of dopaminergic neurons (TUj1, NURR1, LMX1B, TH and PAX6) after 30 days of culture. In conclusion, the authors confirmed that guggulsterone-loaded microspheres promoted differentiation of NPCs into brain cell types.

Table 5 reports Examples of bioprinted models of brain tissue described in the paragraph.

#### 3.2.3. Decellularized Scaffolds for Brain Modeling

Enhanced differentiation of iPSCs into myelin-expressing oligodendrocytes was obtained by their culture on decellularized human brain tissue [246]. Rat-brain-derived decellularized scaffolds functionalized with basic FGF have been developed to study PD [247].

Solubilized decellularized ECM has also been used as functionalizing biomaterial in scaffolds. Electrospun genipin-crosslinked gelatin scaffolds combined with 1% rat ECM [248] were found to induce the differentiation of mesenchymal stem cells (MSCs) towards the neural pathway. Electrospinning was also employed in another study for the fabrication of scaffolds with random and aligned nanofibers, based on PLGA blended with decellularized porcine cauda equina. The resulting scaffold favored the proliferation and the orientation of Schwann cells derived from the sciatic nerve [249].

Other examples include decellularized ECM-based scaffolds employed for regenerative purposes, which have the potential to support the culture of neural and/or glial cells, and could also be applied in brain modeling. In detail, decellularized porcine spinal cord and urinary bladder injectable hydrogels were proven to be efficient in the stimulation of neovascularization and axonal growth in an in vivo model of spinal cord injury (SCI) [250], while decellularized human meningeal scaffolds were employed as 3D platforms supporting the differentiation of hNPCs after SCI [251].

Table 6 Examples of decellularized ECM-based scaffolds described in the paragraph. Despite the advantages linked to the use of such scaffolds, limitations include the need to standardize the preparation protocols employed, the poor availability of human decellularized ECM, and the use animal-derived decellularized ECM, which does not properly recapitulate the human ECM.

#### 3.2.4. Engineered Porous Scaffolds for 3D Culture and Brain Modeling 

Recently, Ranjan et al. [252] developed a novel 3D model of AD by culturing patient-derived iPSC-NPCs in PLGA fibrous scaffolds, prepared by wet electrospinning and coated with laminin. They found increased neural differentiation and reduced cell proliferation after 7 days of culture. Differentiated neurons showed increased levels of pathogenic amyloid-beta 42 (Aβ42) and phospho-tau levels, spontaneously induced by the 3D culture system. The authors concluded that this could be a useful 3D model for recapitulating the pathogenesis of AD.

**Figure 6:**
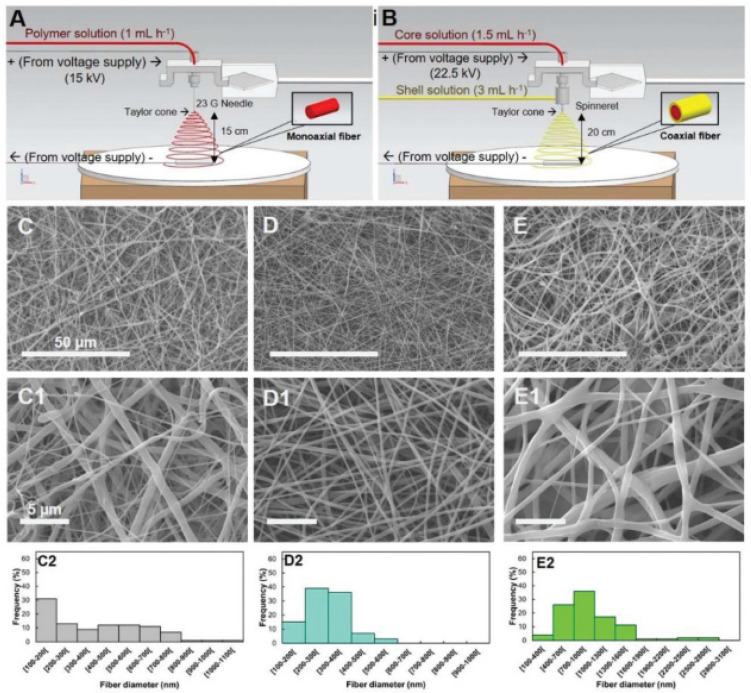
Schematic representation monoaxial (**A**) and coaxial (**B**) fiber production. PCL (**C**,**C1**), PCL-PANI (**D**,**D1**) and PGS/PCL-PANI 13% electrospun fibers (**E**,**E1**), and respective histograms (**C2**,**D2**,**E2**). Reproduced with the permission of Garrudo et al. [253].

Garrudo et al. [253] prepared a 3D fibrous model of brain tissue based on coaxial electrospun fibers, with a soft core layer in the pyrolytic graphite sheet (PGS), in combination with an electroconductive layer made with PCL-PANI (Figure 6). The scaffolds were stable and biodegradable over 21 days, and the effects of the electrical stimulation on iPSC-NPCs were evaluated. The authors found increased expression of neural markers (MAP2) and genes related to excitatory pathways (glutamatergic and voltage-sensitive channel genes), in conjunction with GABAergic marker downregulation, suggesting neuron excitatory activity.

Revkova et al. [254] recently studied the behavior of directly reprogrammed NPCs on electrospun spindroin-based scaffolds enriched with extracellular matrix motifs (RGD, IKVAV and VAEIDGIEL). The authors found that the presence of such motifs favored neuroglial differentiation and, at the same time, about 30% of the progenitor cells were preserved. They concluded that this model could be suitable for the creation of a neuroglial stem cell niche and for controlling their differentiation.

Hsu et al. [255] proposed the use of nature-based fibrous scaffolds for the bioengineering of neural tissue. They employed the electrospinning technique for the fabrication of a fibrous scaffold based on serum albumin (SA), a natural protein with different advantages such as low cost, availability, simplicity of isolation and capability of binding different cellular receptors [256,257]. Moreover, hemin was incorporated into the scaffolds to provide them with conductive properties [258], while recombinant proteins and growth factors were added to induce cell adhesion and proliferation. The scaffolds were prepared by electrospinning SA solution containing 2-mercaptoethanol and doped with hemin. Scaffolds were then coated with laminin and functionalized with FGF2. Laminin coating was retained for at least 3 weeks. Additionally, doped SA mats increased hiPSC-NSC viability after 24 h of culture compared to non-doped scaffolds. Efficient differentiation of cells into neurons occurred after 7 days of culture, as revealed by immunostaining of NSCs for β-III-tubulin. This biomaterial combination enhanced neural maturation of iPSC-NSCs.

Mohtaram et al. [259] prepared scaffolds by the sequential electrospinning of PCL and PCL-RA with different topographies (loop mesh and biaxial), and tested their effects on iPSC differentiation. PCL functionalization with RA increased iPSC differentiation into neural phenotypes, in agreement with previous findings [260,261]. iPSC-NPs were seeded on PCL/PCL-RA scaffolds, and cell viability and differentiation were evaluated after 12 days of culture. The authors showed that PCL/PCL-RA nanofibers supported cell adhesion, and both biaxial and bimodal scaffolds promoted iPSC differentiation into neurons, evaluated by the expression of the neuronal marker β-III-tubulin. Moreover, both scaffold topographies were able to guide neurite outgrowth of human iPSCs, and cells cultured on biaxial scaffolds showed the maximum neurite length.

The electrospinning technique was also used to realize bioartificial scaffolds of polylactic acid (PLA)/gelatin [262]. PLA characteristics make it suitable for use in regenerative medicine, as it is thermoplastic, biocompatible and biodegradable [263]. Gelatin is a natural polymer that derives from the denaturation of collagen, it is non-cytotoxic, non-immunogenic and it is a biomimetic protein that favors cell adhesion [264]. PLA and gelatin solutions were mixed in a common solvent, under magnetic stirring and, then, the solution was electrospun. Scanning electron microscopy (SEM) analysis showed the uniform distribution of gelatin domains into the PLA fibrous matrix structure. After the preparation of the nanofibers, iPSC-derived embryoid bodies (EBs) were cultured on the scaffolds using specific media with differentiating factors, that were basic FGF and NGF for 21 days. Cells showed good interactions with the electrospun structure, which also favored iPSC differentiation into neurons (expression of β-III-tubulin and MAP2). Cell viability was evaluated after 1, 3, 5 and 7 days of culture on PLA/gelatin scaffolds, showing a significant increase on PLA/gelatin scaffolds compared to under control conditions at day 5 and 7. These results demonstrate that PLA/gelatin scaffolds are promising for sustaining iPSCs differentiation.

Mahdi et al. [186] reported, for the first time, the combination of PCL and gelatin to obtain bi-electrospun nanofibers for hiPSCs. Because of its hydrophobicity, PCL can negatively influence cell growth and adhesion, but the addition of gelatin improves cell behavior [265,266]. Bi-electrospun nanofibers were realized by electrospinning technique and then characterized for porosity, pore size and tensile properties. hiPSCs were seeded on scaffolds in the form of EBs and maintained in culture for 14 days. PCL/gelatin scaffolds showed higher hydrophilicity than PCL scaffolds and reduction in porosity and pore size, while Young modulus increased and elongation at break reduced compared to PCL scaffolds. Cell viability was enhanced after 1, 3 and 5 days of culture on PCL/gelatin scaffolds, while real-time PCR and immunocytochemistry analyses confirmed the differentiation into neurons, astrocytes and oligodendrocytes, through the evaluation of markers as GFAP, β-tubulin-III, neuron-specific enolase (NSE), MAP2 and Olig2. These results suggest that fibrous scaffolds based on synthetic and natural polymers represent promising platforms for iPSCs differentiation into brain cell types and the design of in vitro brain models.

Scaffolds can also be produced via the porogen leaching technique. Significant progresses in brain TE models were achieved by Kaplan’s research group. They produced a porous scaffold based on silk proteins for the development of a neurological tissue model using human iPSCs [267]. One of the main advantages of this model concerns the possibility of bypassing early neural differentiation steps (EBs and neural rosettes) favoring the direct integration of iPSCs into the 3D construct. Moreover, the authors combined the porous silk fibroin scaffold, capable of diffusing nutrients and forming networks, with collagen type I, to resemble the extracellular environment of cells and to provide a stable culture system over-time. After silk solubilization and generation of porous scaffolds via salt leaching, the resulting structures were coated with poly-L-ornithine (PLO) and then with laminin. iPSCs were seeded on these scaffolds, which were filled with collagen type I after 5 days of cell expansion. After gelation, scaffolds were flooded with a specific medium, which was changed every 4 days. In addition to cell adhesion, favored by coating with PLO and laminin, cells showed the expression of neural markers MAP2, enolase-2 (ENO2) and β-III-tubulin compared to 2D cultures at the corresponding time points. Moreover, GFAP levels increased, indicating the differentiation of iPSCs into astrocytes, while neurons were healthy and functioning, as demonstrated by electrical measurements. The authors observed that both iPSCs from healthy and AD affected patients were able to differentiate into the 3D scaffolds. This model can be used to study various neurological diseases and their progression.

**Figure 7:**
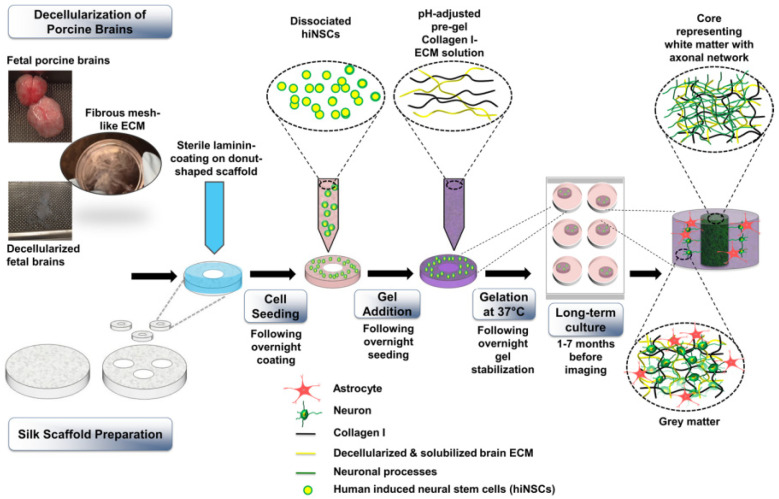
Silk scaffold preparation and human induced neural stem cells (hiNSCs) cultured on bioengineered constructs. From Sood et al. [268].

A later study, published by Sood et al. [268], described a 3D brain model prepared using an SF-based porous scaffold and collagen type I, and the hydrogel was combined with decellularized porcine ECM (Figure 7). According to the authors, SF scaffolds are advantageous as they allow for long-term cultures and the segregation of neural cell bodies, and provide a high surface area for cell attachment. Decellularized porcine ECM was chosen because, in addition to mimicking the brain composition, it contains signals that guide neural organization into microdomains during development [269] and are fundamental for differentiation of neural progenitors and stem cells [270]. Moreover, some ECM proteins are conserved through different species, and porcine-derived ECM is highly biomimetic of human brain ECM [271]. The authors tested the effects of both fetal and adult porcine ECM on hiNSCs, which were obtained after direct reprogramming of dermis-derived human fibroblasts. After decellularization of porcine brain and silk scaffold preparation, cells were seeded on the porous scaffolds, which was then functionalized with collagen type I and porcine-derived ECM. Due to the introduction of brain ECMs, it was possible to observe the growth of mature astrocytes, which were evident after a 2-month culture period, and the downregulation of CSPGs, which are considered markers of astrogliosis [272]. Fetal brain ECM displayed positive effects in increasing calcium oscillatory activity in neurons and downregulating markers of toxic reactive astrocytes. Moreover, differentiated neurons formed early, followed by astrocytes. Cells differentiated on these scaffold types showed spontaneous activity at 7-month versus 3-month culture periods. These characteristics allow the use of this model to investigate neurodegenerative disorders involving astrogliosis. Such findings are considered important to design brain models that favor long-term maintenance of 3D cultured cells.

One of the most recently published models employing SF scaffolds was published by Rouleau et al. [273]. The authors prepared SF porous scaffolds by extraction from silk worm cocoons (*Bombyx mori*), realizing toroidal sponges with a silk-free central window. Then they seeded NPs differentiated by iPSCs and embedded in collagen type I. Scaffolds were maintained in neural media to promote cell growth and differentiation. iPSCs were obtained from both healthy donors and patients suffering from a sporadic form of Alzheimer’s disease. Cell viability assay revealed that cells migrated to the center (hydrogel) of the structure, while the expression of glial markers (GFAP) occurred in long-term cultured samples. Moreover, evaluation of neurodegenerative markers and electrophysiological characterization showed that this model could remain structurally and functionally stable in culture for over 2 years. The cellular microenvironment had a homeostatic role, as revealed by the stable expression of cell stress and neurodegenerative markers in long-term cultures.

Table 7 collects examples of engineered porous scaffold models described in this paragraph.

## 4. Discussion

The progressive aging of the population and increased incidence of age-related diseases, such as neurodegenerative diseases, illustrates the urgent need to find effective treatments. In attempting to achieve this goal, millions of experimental animals are used each year in the EU for preclinical experimentation in basic and applied research regarding the neuronal field [274]. However, limitations of 2D cell cultures and animal models in predicting safety and efficacy of new therapies is responsible for the high percentage of unsuccessful therapies during clinical trials [275]. Hence, the discovery of new effective treatments against brain diseases requires long time periods, high costs and is associated with ethical concerns for the use of a high number of experimental animals. In vitro models of human tissues may help in improving preclinical research towards safe and effective therapies, developed over shorter time scales, at reduced investments and using a lower number of experimental animals.

Examples of such models include TE models of brain tissue, which provide several advantages compared to other 3D models as outlined in Figure 1. Furthermore, iPSCs are now the most preferred cell type for in vitro brain modeling as they are easily available in large quantities, can be differentiated into each brain cell type (both neurons and glial cells) and can be derived from patients, allowing the design of personalized models.

The culture of iPSC-derived NPCs on engineered scaffolds, under additional stimulation with bioactive and soluble molecules, may support the in vitro development of human brain models. Table 8 summarizes the main scaffold requirements for brain tissue engineered models in terms of properties, cell types, characterization and validation. TE scaffolds should provide biomimetic chemical, mechanical and architectural properties in order to properly stimulate iPSC differentiation into brain cells. However, scaffolds based on natural polymers have limited stability in water environments due to their hydrophilicity, leading to high swelling and rapid degradation through hydrolysis, providing time-limited support to cells. Additionally, being extracted from plants or animal tissues, natural polymers suffer from batch-to-batch variability: this specific drawback can be overcome using natural polymers produced by DNA-recombinant technologies; however, these are generally expensive. The combination of natural and synthetic polymers by blending or surface functionalization approaches (also known as “bioartificial materials”) has been widely proposed to generate biomaterials with cell adhesive properties, limited swelling ability, water stability and slow progressive degradation during long-term in vitro cultures (which may be needed for stem cell differentiation). Furthermore, the combination of natural and synthetic polymers is advantageous for the preparation of specific and stable scaffold architectures that are able to provide biomimetic topographical cues to cells, favoring their differentiation. Nanofibrous substrates are particularly promising for in vitro brain modeling and their aligned structure can enhance neuronal differentiation of iPSCs.

Artificial biomaterials could be very promising for 3D brain modeling. Indeed, based on bioartificial materials, the most interesting and promising scaffold architecture for brain modeling is represented by donut-shaped porous scaffolds [267,268,273], produced by Kaplan’s research group. The novelty and the efficiency of such scaffolds depends on both their geometry, which facilitates nutrients diffusion and cell networks formation, and the presence of collagen, which contributes to recreate the ECM environment and to provide a more stable culture system over time. Importantly, the 3D system promotes the co-differentiation of neurons and astrocytes, eliminating the preliminary differentiation steps typically needed with other stem-cell-derived neural tissue models. For all these reasons, we speculate that similar scaffolds represent the most cutting-edge frontier for 3D brain modeling and the most advanced models in the field. Despite these impressive results apported in this research field, the introduction of bioartificial materials to Kaplan’s models could bring a finer modulation of composition, stiffness, architecture and degradation rate, improving their potential towards personalized applications. Moreover, Kaplan’s models do not use a dynamic culture system, which could improve neural differentiation and minimize the effect of the substrate and also lack of endothelial cells and microglia, so they are not representative of the entire brain complexity.

To overcome these limitations, since the brain tissue is based on electrical stimulation, the presence of external or internal electrical stimuli could improve into the design model. Different stimulation platforms to be applied to in vitro model/organ-on-chip system are under investigation, especially for nervous and cardiac tissues [276]. Furthermore, electrically conductive biomaterials [188] have been shown to support neuronal cell differentiation. In this regard, the inclusion of electrically conductive materials, such as CNTs and PPy into the scaffold structure has been found to enhance the differentiation towards the three neural lineages (neurons, astrocytes and oligodendrocytes), as shown by Shin et al. [183]. However, the use of conductive inorganic materials could adversely affect cellular function from a mechanical perspective.

For this reason, the development of platforms based on biodegradable polymeric materials, which are able of promoting cell growth by electrical stimulation, represents a major advance in regenerative medicine of nervous tissue. Moreover, conducting polymers are also used in drug delivery: drugs are bond in the scaffolds and released through an electrical signal. Hence, the development of platforms combining conductive polymers and other materials (natural or synthetic) could improve brain regenerative capacity, in addition of being a valuable model for drug testing.

Despite biomimetic scaffold design being a well-known requirement in brain TE engineering, iPSC differentiation into brain cell types, such as neurons, astrocytes, oligonucleotides and microglia, in their natural proportions, has not been achieved so far. Indeed, in most 3D neural tissue models, neurons and astrocytes have been generated by iPSC differentiation, while oligodendrocytes and microglia have thus far been primarily absent. However, microglia are fundamental for neuron maturation in engineered models [277]. they produce growth factors and anti-inflammatory cytokines, which are beneficial for other brain cell types. On the other hand, issues related to their use in co-culture still remain. For example, the type of culture substrate as well as cell–cell interactions might trigger the activation of an inflammatory phenotype by iPSC-microglia. Furthermore, the design of substrates and the adoption of culture conditions able to properly support co-cultures of multiple cell types is challenging.

Another issue derives from the need to develop an appropriate method for seeding cells into the scaffolds avoiding inflammatory responses or cell detachment. One of the best solutions has been recently found by Bassil et al. [278]. They obtained differentiated neurons from iPSC and then realized a co-culture with primary astrocytes. This system was at the end enriched with microglial cells, obtained after differentiation from iPSC. The authors generated a long-term culture platform for neurons, astrocytes and microglia and used it as a model for AD.

Raimondi et al. [279] used hydrogels comprised of collagen, hyaluronic acid and PEG and prepared an embedded co-culture of neurons and glial cells, previously cultured separately.

In another model [280], iPSCs were cultured in 3D pre-cast gradient hydrogels and differentiated towards excitatory neurons, while primary glial cells were added at day 2, and the functional analysis was performed at day 21. The aim was to obtain mature cells to test drugs for age-related diseases, such as AD.

The above-reported examples suggest that robust 3D brain cell cultures and brain disease modeling may be achieved by separate culture of the different brain cell types (inducing their differentiation separately) followed by their combination. Alternatively, it could be possible to induce iPSC differentiation towards neurons on scaffolds, then adding other differentiated cell types.

The inclusion of oligodendrocytes within brain-tissue-engineered models remains difficult. Recently, Nazari et al. [281] showed enhanced iPSC differentiation into oligodendrocytes within fibrin scaffolds, while Patel et al. [282] demonstrated the ability of hippocampal stem/progenitor cells to differentiate into neurons, astrocytes and oligodendrocytes on PCL microfibers scaffolds. Flagelli et al. [283] reported the efficient differentiation of NPCs into neural lineages, including oligodendrocytes, in a 3D culture system based on polybutylene terephthalate (PBT) fibers functionalized with laminin and observed accelerated maturation of OPCs into OLs with myelin-like morphology. The inclusion of oligodendrocytes in tissue-engineered models could be facilitated by the design of novel functionalized biomaterials.

Due to brain heterogeneity, TE models of specific brain areas should be addressed reproducing their specific ECM features and cell connections.

The modeling of brain pathological conditions requires the knowledge of impaired cell ratios and ECM composition in diseased areas, to be reproduced in engineered models.

Furthermore, in the field of in vitro models, their validation as a preclinical testing tool is extremely important. Typically, 3D in vitro models are validated through viability and morphology studies, often in combination with electrophysiology and microscopy analysis [91]. In this regard, the key parameters to be verified are represented by neuronal outgrowth, cell–cell interactions and neural electrical activity. The last feature is validated through patch clamping and calcium imaging for the assessment of ion flux and electrical conductance. In addition, the evaluation of endothelial cell function can be performed through transepithelial/transendothelial electrical resistance (TEER) technique, that measures membrane permeability and thickness in case of BBB models [284]. Another fundamental validation procedure is the use of drugs already approved for human use and which clinical effect is known, to verify the human brain model response.

Once the TE models have been validated, standardized protocols for their use in in vitro studies should be developed with a preference for non-destructive live monitoring approaches. Some of these include Raman spectroscopy, which is ideal for monitoring cells, substrates and physical relevant metabolites, and two photon excitation microscopy, which represents an interesting alternative to confocal microscopy and also eliminates the need for sectioning 3D engineered constructs [285]. It is also possible to use electrochemical impedance spectroscopy (EIS), which provides information about cell adhesion, proliferation and differentiation over time and it is also used for monitoring TEER [286].

Finally, reproducibility, scalability and simplicity are key targets in the engineering of in vitro models of human brain tissue favoring its widespread use.

**Table 8:** Main requirements of TE human brain models derived from state-of-the-art analysis.

3D SUBSTRATE PROPERTIES		
Scaffold Properties	Target Characteristics	Ref.
Architecture	Fibrous scaffold architecture embedded into a soft hydrogel	[267,268,273]
Composition	Fibrous scaffold based on a synthetic polymer (e.g., PCL, PLGA) or natural polymer (silk fibroin). Hydrogel filler based on: Collagen type I Functionalizing molecules: Laminin, poly-L-ornithin	[267,268,273]
Stiffness	Tailored by composite scaffold composition (target value: brain tissue stiffness of 0.5–14 kPa).	[267,268,273]
Electrical conductivity	Optimal electrical conductivity: 3 × 10^−4^ to 6 × 10^−2^ S/cm	[267,268,273]
Porosity	Optimal porosity: 84–98% range.	[287]
Degradation time	At least a few months to allow construct maturation and further experiments	[288]
**CELLS**		
**Cells**	**Optimal cell Culture Procedure on Scaffolds**	**Ref.**
hiPSC	hiPSC differentiation into some of the brain cells directly on the scaffolds (e.g., neurons and astrocytes), followed by the addition of other cell types.	[278,280]
**MODEL CHARACTERIZATION AND VALIDATION**		
**Model properties**	**Validation of the Model**	**Ref.**
Cell population	Stem cell and differentiation markers expression by PCR, immunofluorescence and Western blot analysis.	[267,268,273]
Brain structure recapitulation	Cell morphology and cell–cell assembly by immunofluorescence analysis.	[267,268,273]
ECM	Characterization of decellularized brain ECM through liquid chromatography–mass spectrometry (LC–MS); GAGs compositional analysis through fluorescence-assisted carbohydrate electrophoresis (FACE).	[267]
Functionality	Functional validation of the model by ion flux and electrical conductance analysis.	[267,268,273]
Predictivity	Predictivity using model drugs, already approved or tested in the clinics.	[267]

## 5. Conclusions

The need for new effective treatments against neurodegenerative diseases is urgent due to the growing incidence of these diseases in the global population. In this regard, in vitro models able to recapitulate pathological conditions for predictive drug testing have been developed, benefitting from new advancements in the field of regenerative medicine.

This review paper provides an extensive overview of the field of in vitro brain models, with a focus on 3D tissue-engineered brain models that enable cell control through a combination of biomimetic scaffold properties and culture conditions. Among available cells, iPSCs are the most promising as they provide a potentially unlimited source of brain tissue-specific cells. Furthermore, patient-specific iPSCs allow the development of personalized in vitro models. The choice of biomaterials and scaffold design is fundamental for guiding and directing cell differentiation to obtain more predictive 3D human brain models. In this regard, “bioartificial” scaffolds based on synthetic polymer fibrous structures embedded into a cellularized natural hydrogel could support long-term cell cultures. Biomimetic cues to be integrated into scaffolds include brain-like stiffness (provided by the natural hydrogel), fibrous architecture (provided by the synthetic polymer fibrous structure) and tissue-like biochemical properties (again provided by the natural polymer, and/or additional functionalizing molecules, such as peptides and growth factors).

The differentiation of iPSC cultured on scaffolds into functional and mature brain cells appear challenging, as different culture conditions are required for the differentiation into the specific brain cells, furthermore cell–cell and cell–substrate interactions may also affect the differentiation outcomes. Recently published reports demonstrated the possibility to co-culture different brain cell types, previously differentiated in monoculture conditions and only later combined. Alternatively, the incorporation of iPSCs into the hydrogel compartment, followed by their differentiation into neurons, and the addition of other previously differentiated glial cells, could be an alternative strategy for the design of in vitro brain models. However, the development of in vitro brain models populated with all the main relevant brain cells, such as neurons, oligodendrocytes, glial cells and astrocytes, is still a challenge, and is limited to only a few reports. In summary, the introduction of 3D models has made a significant improvement compared to 2D models in terms of control of cell fate and behavior. The final aim, hopefully, concerns the possibility to best recreate the complex cellular environment of nervous tissue and to overcome the limitations associated with 2D culture conditions and differentiation protocols.

Once designed, in vitro models should be accurately validated, taking into account the analysis of neuronal growth, electrical activity and cell–cell interactions, and preferentially monitored with non-invasive approaches for a superior high-throughput approach.

Improvement in 3D brain modeling is expected to enhance the phase of preclinical testing of therapies, and to improve the discovery of new treatments for brain diseases. Interestingly, although it is not the scope of this review paper, in silico models could be used in combination with in vitro experimental models on the route towards developing new effective treatments for brain diseases.

## Figures and Tables

**Table 1:** Main cells in the brain tissue.

Cell Types	Characteristics and Functions	References
Neurons	Processing, information storage and transmission of communication signals through chemical or electrical synapses.	[3]
Astrocytes	Involved in neurogenesis, synaptogenesis and in the control of the extracellular homeostasis. Through the astrocyte–neuron lactate shuttle, astrocytes provide energy to neurons and regulate Ca^2+^ concentration, which is fundamental for neuronal activity. Communicate with brain microvascular endothelial cells and pericytes by their endfeet, and contribute to selective transport of ions and water across the blood–brain barrier.	[8,9,10,11]
Oligodendrocytes	Myelinating glia of the CNS. Sustain axon metabolism. Contribute to neuroplasticity.	[12,13,14]
Microglia	Main innate immune cells of the CNS. Crucial for immune response, neural development and function, including neuronal apoptosis, neurogenesis and myelinogenesis.	[15,16,17]
NG2	Characterized by NG2 expression, branched morphology. Distributed through grey and white matter. Important role in remyelination. Interaction with neurons, resulting in the reception of synaptic impulses and in axonal growth contribution.	[5]
Oligodendrocyte precursor cells	Highly proliferative group of progenitor cells. Responsible for oligodendrocyte generation. Monitoring of surrounding environment. Involved in inflammatory responses.	[6,7]

**Table 3:** Main characteristics of 3D platforms for brain tissue engineering.

3D Platform	Characteristics	References
Hydrogels	Hydrophilic networks with outstanding physical and chemical properties. Maximum flexibility and ease in modifying material characteristics. Positive influence on physical guidance and molecule incorporation for localized release.	[195,196]
3D bioprinting	Bioinks loaded with cells and deposited layer-by-layer, obtained with scaffold-based or scaffold-free approaches. Cell differentiation can occur at pre-printing (1) or post-printing (2) stages. Drawbacks include: long time period needed for cell differentiation, inability to control the relative distribution, effects on cell viability.	[197,198,199,200,201]
Decellularized scaffolds	Obtained after removal of cellular components from tissues or organs with different chemical, biological and mechanical methods employed. Advantages include recellularization and cell remodeling due to ECM structure (low immunogenicity and biologically recognizable).	[202,203,204,205,206]
Fibrous scaffolds	Tight control over fiber orientation, high surface-to-volume ratio, effect on cell adhesion, proliferation and differentiation. Produced with techniques such as self-assembly, template synthesis, phase separation, electrospinning.	[207,208,209]

**Table 4:** TE models of human brain tissue based on hydrogel scaffolds.

Main Hydrogel-Based Brain Models					
Materials	Scaffold Type	Cells Used	Culture Times	Main Outputs	Ref.
Alginate/γ-PGA with TATVHL peptide	Inverted colloidal crystal scaffold.	iPSCs	7 days	High cell viability (90%). iPSC differentiation into neurons (βIII tubulin expression).	[210]
HAMA	Density gradient multilayer polymerization technique.	iPSC-NPCs; NPC-derived neurons and astrocytes	6 weeks	Favored neural over glial cells differentiation. Accelerated neuron maturation compared vs. 2D cultures.	[218]
Cathecol-functionalized HA with CNTs and PPy	Oxidative polymerization of cathecol-functionalized HA and in situ PPY polymerization and CNT incorporation.	hfNSCs and hiPSC-NPCs	7 days	hfNSCs differentiation into neurons, astrocytes and oligodendrocytes at 5 days. hiPSC-NPCs differentiation into neurons at 7 days.	[183]
COL-CS; COL-DAC	(1) COL-CS: multiple freeze drying steps followed by EDC crosslinking. (2) COL-DAC: DAC preparation by cellulose oxidation, fabrication of 3D COL sponge shapes and crosslinking in a DAC-containing solution	hiPSC-NPCs	6 days	High proliferation and viability of hiPSC-NPCs. Differentiation into neural cells, astrocytes and oligodendrocytes.	[185]
COLMA/HAMA/ALGMA with GRGDSP and Ln5-P4.	Photocrosslinking using a mask.	iPSCs	3 days	iPSC neural differentiation increased to 98% after induction by NGF	[182]
Laminin-coated PEGDA; control TMPTA	PolyHIPE scaffolds	hiPSC-NSCs	14 days	Upregulation of glial cell markers especially on TMPTA scaffolds; Increased spontaneous calcium activity within laminin-coated PEGDA scaffolds.	[228]
PAAM-CH, PLGA NPs	Inverted colloidal crystal scaffolds	iPSCs	3 days of culture	Enhanced neural differentiation	[187]

**Table 5:** Main TE models of human brain tissue based on bioprinting technologies.

Bioprinted Models of Brain Tissue					
Bioink Hydrogel	Bioprinting Technique	Cells Used	Culture Times	Main Outputs	Ref.
Alginate (5%), carboxymethyl chitosan (5%) and agarose (1.5%)	Microextrusion bioprinting	hNSCs	10 days	Differentiation of hNSCs into neurons and glial cells post-printing.	[234]
As above	Microextrusion bioprinting	iPSCs	10 days in proliferative medium; 11–20 days in a differentiation BDNF-containing medium	Neuronal and glial cells differentiation with spontaneous and bicuculline-induced calcium responses at 20 days.	[236]
Matrigel and alginate (1:1 weight ratio; 2% alginate concentration	Microextrusion bioprinting	iPSCs differentiated into cortical neurons	1, 7, 50 and 70 days.	Expression of neuronal (TRB1 for mature cortical neurons) and glial (GFAP) markers, and calcium activity at 7 days. Mature cortical neurons were maintained in neuronal differentiation medium up to 70 days.	[237]
Fibrinogen, alginate, chitosan, calcium chloride, thrombin and genipin.	Microextrusion bioprinting and lab-on-a-printer technology	hiPSC aggregates	41 days: at 17 days addition of RA to induce differentiation into dopaminergic neurons	Expression of Tuj1 (an early neuronal marker) at day 41 by immunostaining.	[240]
As above with addition of guggulsterone-loaded microspheres to promote cell differentiation.	Microfluidics-based RX1 bioprinter	hiPSC-NPCs	Up to 30 days	At 15 and 30 days: neural markers detected by immunostaining; cells expressing glial (GFAP) and oligodendrocyte markers (O4) assessed by flow cytometry. At 30 days: expression of dopaminergic markers (TUj1, NURR1, LMX1B, TH and PAX6).	[243]

**Table 6:** Decellularized ECM-based TE scaffolds for brain modeling or supporting neuronal and glial cells.

Decellularized ECM-Based Scaffolds for Brain Modeling or Supporting Nerve/Glial Cells					
Substrate	Other Stimuli	Cells Used	Culture Times	Main Outputs	Ref.
Decellularized human brain tissue	Co-culture with mouse induced neurons (iN)	iPSC-OPCs	14 days	iPSCs differentiated into myelin-expressing oligodendrocytes	[246]
Decellularized human brain tissue	Functionalized with basic FGF	PC-12	24 h	In vitro PD model	[247]
Electrospun genipin-crosslinked gelatin scaffolds with 1% rat ECM	**-**	MSCs	7 days	Differentiation of MSCs towards neural cells	[248]
Electrospun PLGA blended with decellularized porcine cauda equina	**-**	Schwann cells derived from sciatic nerve	7 days	Scaffolds favored the proliferation and the orientation of Schwann cells	[249]
Decellularized porcine spinal cord and urinary bladder injectable hydrogels	**-**	hWJ-MSCs	7 days	Stimulation of neovascularization and axonal growth in an in vivo model of spinal cord injury	[250]
Decellularized human meningeal scaffolds	**-**	hNPCs	21 days	Differentiation of hNPCs	[251]

**Table 7:** TE models of human brain tissue based on other TE technologies.

Main TE Models of Brain Tissue Reported in the Literature					
Materials	Substrate	Cells Used	Culture Times	Main Outputs	Ref.
PLGA	Wet electrospun fibrous scaffolds	iPSC-NPCs	7 days	AD model: neuronal differentiation with pathogenic Aβ42 and phospho-tau levels	[252]
Soft core layer in pyrolytic graphite sheet (PGS), combined with an electroconductive PCL-PANI layer.	Coaxial electrospun fibrous scaffolds	iPSC-NPCs	21 days	Increased expression of neural markers (MAP2) and genes related to excitatory pathways (glutamatergic and voltage-sensitive channel genes). Downregulation of GABAergic markers.	[253]
Spindroin-PCL enriched with extracellular matrix peptide motifs (RGD, IKVAV and VAEIDGIEL).	Electrospun scaffolds with aligned fibers	Directly reprogrammed NPCs	Proliferation during first 3 days and differentiation during 4–14 days.	RGD promoted a lower number of neurons with longer neurites; IKVAV supported a higher number of NF200-positive neurons with shorter neurites. Obtainment of neuroglial stem cell niche with preservation of stem cells.	[254]
SA with hemin, laminin coating and functionalization with FGF2	Electrospun scaffolds	iPSC-NSCs	7 days	Neural maturation of iPSC-NSCs assessed by β-III-tubulin expression.	[255]
PCL + PCL-RA	Melt electrospun PCL scaffolds (loop mesh and biaxially aligned microscale topographies), coated with electrospun PCL/RA	iPSC-NPCs	12 days	iPSC-NP differentiation into neurons, evaluated by the expression of the neuronal marker β-III-tubulin. Maximum neurite outgrowth on biaxial scaffolds.	[259]
Polylactic acid (PLA)/gelatin	Electrospun scaffolds	hiPSCs embryoid bodies (EBs)	21 days using media with differentiating factors: FGF and NGF	Expression of β-III-tubulin and MAP2 iPSC suggesting differentiation into neurons.	[262]
PCL/Gelatin	Bi-electrospun nanofibers	hiPSCs embryoid bodies (EBs)	14 days	GFAP, β-tubulin-III, neuron-specific enolase (NSE), MAP2 and Olig2 expression demonstrated differentiation into neurons, astrocytes and oligodendrocytes.	[186]
Silk fibroin porous structure with poly-L-ornithine and laminin coatings and collagen I hydrogel filler	Salt leaching	iPSCs	8 months	Expression of MAP2, enolase-2 and β-tubulin-III demonstrated differentiation into astrocytes and neurons.	[267]
Silk fibroin porous scaffold with laminin coating and collagen type I, plus decellularized porcine ECM as filler	Salt leaching	hiNSCs	7 months	Growth of mature astrocytes, downregulation of CSPGs (marker of astrogliosis after 2-month culture.	[268]
Silk fibroin scaffolds with a silk-free central window and filled with collagen I during cell seeding.	Salt leaching	hiPSC	2 years	Glial marker expression in long-term cultures. Structural and functional stability for over 2 years.	[273]

## Abbreviations

αβ42	Amyloid-beta 42
AD	Alzheimer’s disease
ALGMA	Methacrylated alginate
ASCs	Adult stem cells
BBB	Blood–brain barrier
BDNF	Brain-derived growth factor
BMECs	Brain microvascular endothelial cells
BoC	Brain-on-chip
CMC	Carboxymethyl chitosan
CNS	Central nervous system
CNT	Carbon nanotube
COL	Collagen
COLMA	Methacrylated collagen
CS	Chondroitin sulphate
CSPGs	Chondroitin sulphate proteoglycans
DA	Dopaminergic
DAC	2,3–dialdehyde
dbcAMP	Dibutyryl cyclic adenosine monophosphate
EBs	Embryoid bodies
ECM	Extracellular matrix
ENO2	Enolase 2
ESCs	Embryonic stem cells
FACE	Fluorescence-assisted carbohydrate electrophoresis
GAGs	Glycosaminoglycans
GFAP	Glial fibrillary acidic protein
HA	Hyaluronic acid
HA-CA	Cathecol (functionalized) hyaluronic acid
HAMA	Methacrylated hyaluronic acid
hfNSCs	Human fetal neural stem cells
HSPGs	Heparan sulphate proteoglycans
ICC	Inverted colloidal crystal
iPSCs	Induced pluripotent stem cells
LC–MS	Liquid chromatography–mass spectrometry
LOP	Lab-on-a-printer
MAP2	Microtubule-associated protein 2
MMP	Metalloproteinases
MSCs	Mesenchymal stem cells
NGF	Nerve growth factor
NPCs	Neural progenitor cells
NSCs	Neural stem cells
NPs	Nanoparticles
OLs	Oligodendrocytes
OPCs	Oligodendrocyte precursor cells
OPN	Osteopontin
Pγ	Pyrrole
PAAM–CH	Polyacrylamide–chitosan
PANi	Polyaniline
PBT	Polybutylene terephthalate
PCL	Polycaprolactone
PD	Parkinson’s disease
PEDOT	Poly-3,4-ethylenedioxythiophene
PEGDA	Polyethylene glycol diacrylate
γ-PGA	Poly-γ-glutamic acid
PGS	Pyrolytic graphite sheet
PLA	Polylactic acid
PLO	Poly-L-ornithine
PLGA	Polylactic-co-glycolic-acid
polyHIPE	Polymerized high internal phase emulsions
PPγ	Polypyrrole
RA	Retinoic acid
S100β	S100 calcium-binding protein β
SEM	Scanning electron microscopy
SF	Silk fibroin
SPI	Spinal cord injury
TE	Tissue engineering
TMPTA	Trimethylolpropane triacrylate
TPA	12-O-tetradecanoylphorbol-13-acetate
Tuj1	β-III-tubulin
